# Quantitative investigation of the urinary excretion of three specific monoester metabolites of the plasticizer diisononyl adipate (DINA)

**DOI:** 10.17179/excli2021-3360

**Published:** 2021-02-19

**Authors:** Alexandra Gotthardt, Daniel Bury, Hans-Willi Kling, Rainer Otter, Tobias Weiss, Thomas Brüning, Holger M. Koch

**Affiliations:** 1 Institute for Prevention and Occupational Medicine of the German Social Accident Insurance, Institute of the Ruhr-University Bochum (IPA), Bürkle-de-la-Camp-Platz 1, 44789 Bochum, Germany; 2 University of Wuppertal, Department of Chemistry and Biology, Gaußstraße 20, 42119 Wuppertal, Germany; 3 BASF SE, Industrial Petrochemicals Europe; E-CPI/R-H202, Carl-Bosch-Straße 38, 67056 Ludwigshafen, Germany

**Keywords:** diisononyl adipate, DINA, plasticizer, metabolism, oral dose, human biomonitoring

## Abstract

Diisononyl adipate (DINA) is a plasticizer used in PVC products as an alternative for restricted phthalate plasticizers. With this study, we provide first data on human DINA metabolism and excretion. We postulated mono(hydroxy-isononyl) adipate (OH-MINA), mono(oxo-isononyl) adipate (oxo-MINA), and mono(carboxy-isooctyl) adipate (cx-MIOA) as specific DINA metabolites based on the known human metabolism of structurally similar adipates and phthalates. Urinary excretion was quantitatively investigated after a single oral dose (113 to 145 µg/kg body weight) to three healthy volunteers using a newly developed online-SPE-LC-MS/MS method with isotope dilution and LOQs between 0.3 - 0.6 µg/L. OH-MINA turned out to be the major of the three metabolites with consistent urinary excretion fractions (F_UE_s) of 0.020-0.023 % among all volunteers. Oxo-MINA and cx-MIOA were excreted with lower shares (mean: 0.003 % and 0.009 %, respectively). For all three metabolites, urinary concentrations peaked quickly between 1.4 and 2.3 h post dose with maximum concentrations of 23.1 (OH-MINA), 2.87 (oxo-MINA) and 9.83 µg/L (cx-MIOA). Thus, F_UE_s and urinary concentrations were rather low for these specific metabolites, with the major share of the dose presumably being excreted as non-specific metabolites such as adipic acid. In a pilot population (n=35) of German adults without known DINA exposure, we could not detect any of the three metabolites, contrary to the dosage study, indicating to population exposures lower than 50 µg/kg body weight/day. The new HBM method in conjunction with the new F_UE_s can be used for objective DINA exposure and risk assessment especially in populations with potentially higher DINA exposures.

## Introduction

Due to their endocrine disrupting potency and reproductive toxicity, the use of some ortho-phthalate plasticizers such as di(2-ethylhexyl)phthalate (DEHP) has been strictly regulated in many parts of the world (EC, 1999[14], 2011[12][13], 2018[11]; EPC, 2005[20], 2006[19], 2009[21]; CPSC, 2008[10]; Minister of Justice Canada, 2010[39], 2016[40]). Diisononyl adipate (DINA, CAS registry no. 33703-08-1; EC no. 251-646-7) is an alternative to regulated, high molecular weight phthalates, that is mainly used to achieve low-temperature flexibility in PVC products (BASF SE, 2019[6]; ExxonMobil Chemical, 2016[22]). Consumer near applications include plastic and rubber articles, electrical and electronic products, e.g., wire and cable jacketing, as well as toys and childcare products (Abe et al., 2003[1], 2012[2]; Biedermann-Brem et al., 2008[7]; Maag et al., 2010[38]; IHS Markit, 2018[27]; ExxonMobil Chemical, 2016[22]; US EPA, 2018[56]; ECHA, 2020[18]). A further application of DINA is as an additive in greases and lubricants (ECHA, 2020[18]). DINA may not be used in food contact materials (FCM) in the EU, but in the U.S and parts of Asia it is permitted to be used in FCM in contact with non-fatty and non-alcoholic foods (IHS Markit, 2018[27]; NHFPC China, 2016[44]; FDA, 2019[23]; EC, 2011[12]). 

DINA is produced by esterification of adipic acid with low branched C9-isononanols (BASF SE, 2019[6]) or with a mixture of branched C8-C10 alcohols, containing predominantly C9 alcohols (ExxonMobil Chemical, 2016[22]). Regardless of the manufacturing process (and effectively a different chemical composition), the resulting plasticizer DINA is characterized by the same CAS registry number. The U.S. Environmental Protection Agency refers to DINA as a high production volume chemical (US EPA, 2018[56]). In 2019, 4,500 tons of DINA were consumed in the U.S., while in Western Europe the consumption amounted to 6,000 tons (IHS Markit, 2018[27]). 

Scientific data on the toxicity of DINA is limited and no studies regarding reproductive, developmental, and chronic toxicity are publicly available to our knowledge. The European Chemicals Agency (ECHA), the US EPA, and US Consumer Product Safety Commission (CPSC) reported secondary literature describing subchronic oral dose studies in rats and beagles. In rats even at the highest dose level of 500 mg/kg body weight (bw)/day (d) no adverse effects could be observed, while in beagles a no observed adverse effect level (NOAEL) of 274 mg/kg bw/d was identified based on decreased body weights and changes in livers and kidneys (US EPA, 2018[56]; ECHA, 2020[15][16]; Carlisle et al., 2019[8]). So far, a tolerable daily intake (TDI) for DINA has not been derived. The REACH registration file available from ECHA reports a Derived No Effect Level (DNEL) on the oral route for the general population of 0.85 mg/kg bw/d based on read-across to DEHA, where a NOAEL of 170 mg/kg bw/d was identified in a one-generation study on the basis of reduced body weight gain in maternal animals and offspring (ECHA, 2020[17]).

Exposure of the general population to DINA is likely, considering its consumer applications, including food contact materials (outside the EU), toys, and child care articles. Migration of DINA from PVC cling film into wrapped food has already been described (Saito et al., 2002[45]; Tsumura et al., 2003[55]; Kawamura et al., 2017[29]; Carlos et al., 2018[9]). Based on the DINA content of such cling films and the average food consumption, a daily intake of 21 µg/kg bw/d was estimated for the Japanese population from that exposure source (Kawamura et al. 2017[29]). In Germany, however, DINA has been detected only in 5 % of house dust samples from daycare centers with a maximum concentration of 34 mg/kg, and thus at much lower incidence rates and concentrations than other plasticizers such as DEHP (Fromme et al., 2016[25]). Human Biomonitoring (HBM) provides an integral measure for exposure assessment covering all routes of uptake (oral, inhalation, dermal) and exposure sources and thus can be used for robust exposure and risk assessments (Needham et al., 2007[41]; Angerer et al., 2007[4]; Schindler et al., 2014[46]; Schwedler et al., 2017[49]; Kolossa-Gehring et al., 2017[36]; Haines et al., 2017[26]). We have already presented such an approach for the adipate di(2-ethylhexyl) adipate (DEHA) (Nehring et al., 2020[43]) and similar approaches have been established by us and others for a wide range of phthalate plasticizers and their alternatives such as di(isononyl)cyclohexane-1,2-dicarboxylate (DINCH) and di(2-ethylhexyl) terephthalate (DEHTP) (Zota et al., 2014[58]; Silva et al., 2017[53]; Koch et al., 2017[33]; Schwedler et al., 2020[50][51][48]; Lessmann et al., 2019[37]; Kasper-Sonnenberg et al., 2019[28]; Frederiksen et al., 2020[24]). Thus, the aim of this study was to identify human metabolites of DINA in urine as specific DINA exposure biomarkers, to investigate their elimination kinetics in order to obtain F_UE_s for an objective DINA exposure and risk assessment, and to apply this approach in a small pilot population. 

## Material and Methods

### Chemicals

Diisononyl adipate (≥ 99.5 %) used in the oral dosing study was provided by BASF SE (Ludwigshafen, Germany). The analytical standards of the DINA metabolites 1-mono-(4-methyl-7-hydroxyoctyl) adipate (7OH-MINA; > 97 %), 1-mono-(4-methyl-7-oxooctyl) adipate (7oxo-MINA; > 97 %), and 1-mono-(4-methyl-7-carboxyheptyl) adipate (7cx-MIOA; > 97 %), as well as DEHA metabolites (for simultaneous determination of DINA and DEHA metabolites; see Supplementary material) 1-mono-(2-ethyl-5-hydroxyhexyl) adipate (5OH-MEHA; > 95 % ), 1-mono-(2-ethyl-5-oxohexyl) adipate (5oxo-MEHA; > 97 %), 1-mono-(2-ethyl-5-carboxypentyl) adipate (5cx-MEPA; > 97 %), and their respective ^13^C_6_-labeled analogs (^13^C_6_-7OH-MINA, ^13^C_6_-7cx-MIOA,^ 13^C_6_-5OH-MEHA, ^13^C_6_-5oxo-MEHA, and ^13^C_6_-5cx-MEPA; same chemical purity as corresponding unlabeled chemicals, (labels in the adipic acid moiety) were custom synthesized by Dr. Vladimir Belov (Max Planck Institute for Biophysical Chemistry, Göttingen, Germany). Identities and purities of all labeled and non-labeled standard substances were confirmed by ESI-MS and ^1^H NMR. Water, acetonitrile and methanol (all CHROMASOLV^TM^ LC-MS), ethanol (≥ 99.8 %), and formic acid (puriss p.a.) were purchased from Honeywell (Seelze, Germany). Acetic acid (100 % for LC-MS) was purchased from Merck (Darmstadt, Germany). Ammonium acetate BioXtra (≥ 98 %) was purchased from Sigma Aldrich (Steinheim, Germany). Pure *β*-glucuronidase (without arylsulfatase/esterase activity) from *E. coli* K12 was purchased from Roche Diagnostics (Mannheim, Germany).

### Dosing study

Three healthy German volunteers (2 females, 1 male; aged between 25 and 37 years; body weight between 67 and 86 kg), all without known occupational exposure to DINA, received a single oral dose of approximately 10 mg DINA (weighed precisely), resulting in individual doses between 113 to 145 µg/kg bw, being roughly a factor of 6-8 below the long-term DNEL of DINA of 850 µg/kg bw/d. The DINA dose was dissolved in 1 mL ethanol and diluted with 5 mL water and was provided in a chocolate coated waffle cup. After receiving the DINA dose, the volunteers had a small breakfast. 

Full void urine samples were collected immediately before the dose (t=0), as well as for 48 h after dose in separate 250 mL polyethylene containers and stored frozen at -20 °C until further use. Sampling times were noted by the volunteers and sample volumes were determined by the mass difference between empty and filled sample containers. Urinary creatinine concentrations were determined by L.u.P GmbH Labor- und Praxisservice (Bochum, Germany).

### Pilot population

In addition to the metabolism study, convenience spot urine samples from a small pilot population were analyzed for DINA metabolites. The pilot population consisted of 35 German volunteers (age 23 to 59 (median 42), 24 females and 11 males), not occupationally exposed to DINA. Urine samples had been collected in April 2017. 

### Chemical analyses of DINA (and DEHA) metabolites

The secondary oxidized DINA metabolites mono-(hydroxyisononyl) adipate (OH-MINA), mono-(oxoisononyl) adipate (oxo-MINA), and mono-(carboxyisooctyl) adipate (cx-MIOA) were analyzed using a newly developed online-SPE-LC-MS/MS method, based on a method for DEHA metabolites, previously published by our group (Nehring et al., 2019[42]). The DEHA metabolites 5OH-MEHA, 5oxo-MEHA, and 5cx-MEPA were also included in the method because of shared fragmentation patterns and potential chromatographic overlaps. In brief, 300 µL urine (or calibration solution) were mixed with 100 µL ammonium acetate buffer (1 M, pH = 6.0 - 6.4), 20 µL internal standard solution (^13^C_6_-labeled standards in water), and 6 µL β-glucuronidase (premixed 1:1 with ammonium acetate buffer). After enzymatic deconjugation of glucuronic acid conjugates (2 h, 37 °C), 30 µL formic acid were added and the samples were frozen over night at -20 °C to precipitate cryophobic proteins. Samples were thawed at room temperature, centrifuged (10 min at 1900 g) and 25 µL of the supernatant were analyzed by liquid chromatography (LC) , using a phenylhexyl-modified silica gel column with superficially porous particles (Kinetex® Phenyl-Hexyl 150x3 mm, particle size 2.6 µm; with SecurityGuard™ ULTRA Cartridges UHPLC Phenyl 3.0mm ID Columns; Phemomenex, Aschaffenburg, Germany) for chromatographic separation, coupled with online turbulent flow chromatography for matrix depletion and analyte enrichment (TurboFlow® Phenyl 50 x 0.5 mm; Thermo ScientificTM, Franklin, MA, USA) (online-SPE). Detection was performed by electrospray ionization-triple quadrupole-tandem mass spectrometry (ESI-MS/MS) in negative ion mode using the time-programmed multiple reaction monitoring (scheduled MRM) detection mode. For a more detailed description see Supplementary material. 

### Statistics

Data analysis was conducted with Microsoft Excel 2010 (Microsoft Corporation, Redmond, WA, USA). Urinary excretion fractions (F_UEs_) were calculated, using the following equation (∑m_i_: sum of masses of the respective metabolite in all urine samples, M(DINA): molar mass of DINA, M(Metabolite): molar mass of the respective metabolite, and D the absolute applied DINA dose): 


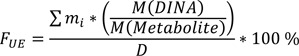


## Results and Discussion

### DINA metabolites under investigation and their quantification

**Figure 1:**
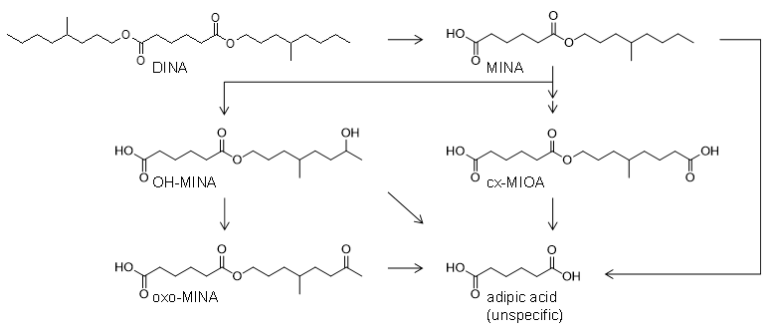
Human metabolism pathways of DINA. The side chain oxidized monoester metabolites OH-MINA, oxo-MINA and cx-MIOA (only isomers based on the 4-methyloctyl side chain shown for simplification) were confirmed and quantitatively investigated in the current study. Furthermore, in analogy to human DEHA and DnBA metabolism, adipic acid (AA) is assumed as the ultimate breakdown product (not investigated in this study). For simplification, phase two metabolites (e.g. glucuronic acid conjugates) are not shown.

The metabolism pathways leading to the postulated oxidized monoester metabolites OH-MINA, oxo-MINA and cx-MIOA, and adipic acid (AA) after full hydrolyses of DINA are shown in Figure 1(Fig. 1) (for simplification, only the 4-methyloctyl based isomers are shown for each metabolite). Based on the knowledge on human DEHA and DnBA (publication in preparation) metabolism, AA will most likely be the ultimate product of hydrolytic DINA metabolism and a major metabolite of DINA. However, AA is not specific for DINA exposure (not even as a sum parameter for adipic acid esters because AA itself is a registered food additive (E 355) and food contact material in Europe (FCM 303)) and high background concentrations were observed in the general population (Nehring et al., 2020[43]; for DnBA publication in preparation). Thus AA was not investigated in this study.

Considering the complexity of the isomeric composition of DINA itself, as well as a variety of possible positions for oxidative functionalization by phase I metabolism, it is not feasible to cover all isomers with individual analytical standards. Instead, we obtained defined analytical standards based on the 4-methyloctyl isomer, which has been shown as a major isomer in industrial isononyl alcohol (INA) (Koch et al., 2007[32]) with oxidative modifications at position 7 (OH- and oxo-MINA) or 8 (cx-MIOA) of the alkyl side chain. For OH-MINA and cx-MIOA, which were expected to be of greater quantitative relevance compared to oxo-MINA based on experiences with DEHA and DnBA (Nehring et al., 2020[43]; for DnBA publication in preparation), we also obtained ^13^C_6_-labeled internal standards. 

**Figure 2:**
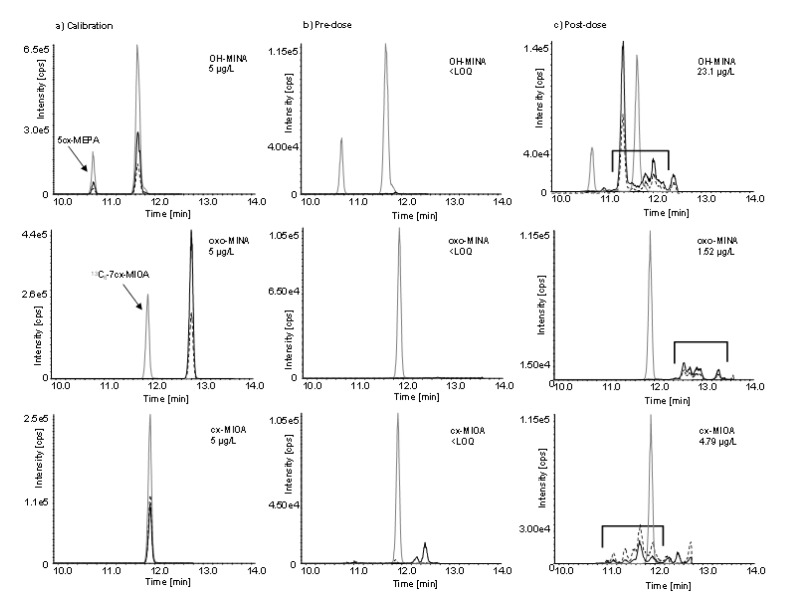
Chromatograms of (a) a calibration standard in water; (b) a urine sample collected before dose (OH-MINA, oxo-MINA, and cx-MIOA below the LOQ; only the internal standard peaks can be seen); and (c) a urine sample collected 2 h after oral dose. Native DINA metabolite traces shown in black (quantifier transitions as continuous lines and qualifier transitions in dotted lines) and labeled internal standard traces in gray. The time frame of elution of the dose related metabolite isomers in (c) is indicated by open brackets

Figure 2(Fig. 2) shows exemplary chromatograms of the three target metabolites in a calibration standard in water (left column), a pre dose, general population urine sample (middle column), and a post dose urine sample (right column). Narrow single chromatographic peaks were obtained for each analytical standard (labeled and unlabeled) due to the single oxidized 4-methyloctyl isomer that was used as a representative of all possible DINA metabolites. No DINA metabolites could be detected in the pre dose urine sample. The two peaks observed in the cx-MIOA traces did not belong to DINA because these peaks did neither increase after the DINA dose nor follow any other observable kinetic. In urine samples after the oral dose, characteristic peak patterns emerged (right column), eluting within a time-frame of roughly 1.5 minutes, reflecting the complex mixture of DINA metabolite isomers. As can be seen, the 4-methyloctyl isomer standards elute in the midst of the isomeric peak pattern, but do not necessarily represent the major peak. Quantification was performed by cumulative integration of all isomers of each metabolite (i.e., OH-MINA, oxo-MINA, and cx-MIOA) and via the calibration curve obtained for the respective monoisomeric analytical standard. Although some of the isomers might not be ideally quantified this way, this type of consensus method approach has already been established for other isomeric plasticizers with isononyl alkyl chains such as diisononyl phthalate DINP (Wittassek et al., 2007[57]; Koch and Angerer, 2007[30]; Koch et al., 2007[32], 2017[33]; Anderson et al., 2011[3]; Frederiksen et al., 2020[24]; Apel et al., 2020[5]) or DINCH (Silva et al., 2013[52]; Koch et al., 2013[34]; Schütze et al., 2014[47]; Kasper-Sonnenberg et al., 2019[28]). Within the isomeric peak patterns, we observed different quantifier/qualifier ratios (up to 2-fold difference) and consequently also somewhat different quantifier/qualifier ratios for the analytical standards on one hand and the respective native metabolites (sum of peak areas of all isomers) on the other hand. This indicated that the exact constitution of the different isononyl isomers, as well as the position of the functional groups has an influence on the fragmentation behavior of the metabolites. However, quantifier/qualifier ratios for the urine samples were within narrow limits for each individual metabolite (again, sum of isomers), despite the differences observed in comparison to the monoisomeric standards. 

Another aspect to be pointed out is that the DINA metabolite OH-MINA and the DEHA metabolite 5cx-MEPA are two isobaric compounds (or mixture of compounds in case of OH-MINA) and OH-MINA shares all its (adipic-acid derived) mass fragments with 5cx-MEPA. Specific side chain-derived fragments were only detected for 5cx-MEPA but not for OH-MINA. Therefore, special attention had to be paid to the chromatographic separation of 5cx-MEPA from the OH-MINA isomers, which could be achieved as can be seen in the post dose chromatogram (right column, upper row) with 5cx-MEPA eluting before the OH-MINA isomeric peak pattern.

The accuracy, determined through spiking of three different concentration levels of the authentic standard substances to eight different urine samples (creatinine content 0.34 to 2.3 g/L) was very satisfactory (relative recoveries 90-121 %) for all DINA and DEHA monoester metabolites. For oxo-MINA, with no authentic internal standard available (^13^C_6_-7cx-MIOA was used as a surrogate internal standard) we determined relative recoveries of 38-112 %, indicating to problems in some urine samples. Excluding one urine sample with recovery issues, relative recoveries in the other seven samples were in a satisfactory range (72-112 %). Nevertheless, we currently regard oxo-MINA results as semi-quantitative. The method's precision was satisfactory for all metabolites with intra- and interday coefficients of variation ≤ 12 % (≤ 7.6 %, excluding oxo-MINA). The limits of quantification (LOQ) (based on a signal-to-noise ratio of 10 in native urine samples containing the target analytes) were 0.3 µg/L (OH-MINA and oxo-MINA), 0.6 µg/L (cx-MIOA), 0.5 µg/L (5OH-MEHA), 0.1 µg/L (5oxo-MEHA), and 0.05 µg/L (5cx-MEPA). For more details, see Supplementary material.

### Elimination kinetics of DINA monoester metabolites after oral dose

After the single oral DINA dose, the three volunteers donated 24, 23, and 29 individual, full volume urine samples with a total urine volume of 3824, 2044, and 3020 mL over the period of 48 hours. In none of the pre dose samples, any of the three oxidized DINA metabolites could be detected. However, they quickly emerged in the post dose samples (see Figure 2(Fig. 2), right column, for exemplary chromatograms) with maximum urinary concentrations (c_max_ see Table 1(Tab. 1), sum of isomers) at least a factor of 55 (OH-MINA), 5 (oxo-MINA), and 8 (cx-MINA) above the respective LOQs.

**Figure 3:**
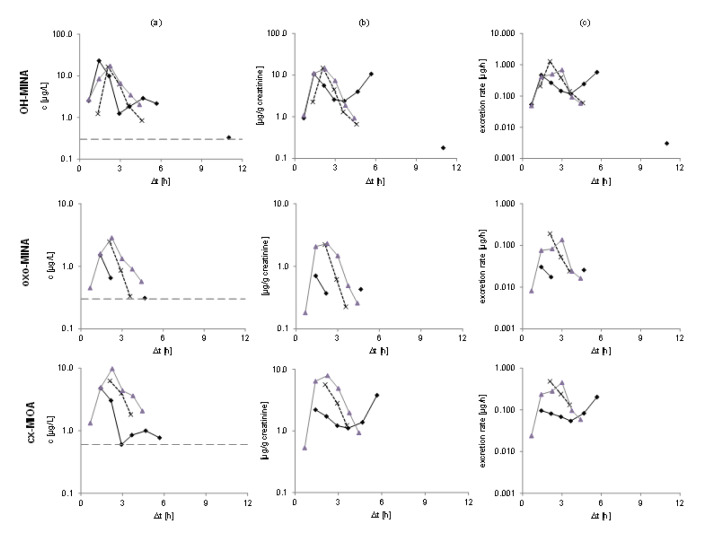
Elimination kinetics of OH-MINA (top), oxo-MINA (middle), and cx-MIOA (bottom) for all three volunteers after oral DINA dose. Unadjusted concentrations in μg/L with respective LOQs shown with gray dotted lines are presented in column (a), creatinine-adjusted concentrations in μg/g creatinine in column (b), and excretion rates in μg/h in column (c).

Figure 3(Fig. 3) shows the urinary excretion kinetics for the three DINA monoester metabolites in all three volunteers. Absolute concentrations (in µg/L, left panel), creatinine-adjusted concentrations (in µg/g creatinine, middle), and the excretion rates (in µg/h, right panel) are shown on a logarithmic scale. For all three metabolites, urinary peak concentrations (c_max - _see Table 1(Tab. 1)) were observed between 1.4 and 2.3 h after oral dose. For one volunteer (depicted with black solid line), a second peak was observed at 5.7 h for OH-MINA and cx-MIOA (oxo-MINA was below the LOQ for this time point).

After the maximum (or maxima, in case of one volunteer), the metabolite concentrations rapidly fell below the respective LOQs in all volunteers after 4.7-5.7 h for OH-MINA (with one single sample from one volunteer above the LOQ at 11 h post dose), 2.2-4.4 h for oxo-MINA (with only one sample above the LOQ at 4.7 h post dose), and 3.6-5.7 h for cx-MIOA. F_UE_s calculated for these three side chain oxidized DINA metabolites based on their total amounts excreted via urine are shown in Table 2(Tab. 2). In line with the peak concentrations observed (Table 1(Tab. 1)), OH-MINA was the major of the three DINA metabolites, accounting for 0.022 % (mean) of the DINA dose. The F_UE_s were very consistent between the three volunteers (0.020-0.023 %). The two other metabolites were excreted at considerably lower dose shares (0.009 % for cx-MIOA and 0.003 % for oxo-MINA).

In addition to the excretion kinetics of the specific DINA metabolites we investigated the glucuronidation patterns. To determine the total amounts (sum of glucuronidated and non-conjugated metabolite) of DINA metabolites, pooled urine samples (0-6 h) from each volunteer were treated the same way as described in section “Chemical analyses of DINA (and DEHA) metabolites”. For the determination of free, non-conjugated metabolites the same procedure without addition of β-glucuronidase was applied. The percentages of non-conjugated ('free') metabolites in relation to the total amounts was negligible (0-7 %) for all three DINA metabolites and volunteers. Due to the strongly non-polar alkyl side chain, conjugation seems necessary to provide needed hydrophilicity for excretion via urine.

### Comparison with other adipate esters

In total, only 0.034 % of the oral DINA dose could be recovered in urine as the above three oxidized DINA monoester metabolites. This percentage is disappointingly low from the perspective of identifying sensitive and specific urinary exposure biomarkers for DINA. However, it is in line with urinary excretion characteristics known for other adipates.

For DEHA, the homologous adipate with shorter alkyl chains by one carbon, the oxidized monoester metabolites were excreted with a sum-F_UE_ of 0.32 % (Nehring et al., 2020[43]). For DnBA, the oxidized metabolites represented 0.49 % (publication in preparation). Thus, these low F_UE_s seem to further diminish with increasing alkyl chain lengths. Because we could show for DnBA and DEHA that the simple monoester was not excreted in relevant amounts via urine (Nehring et al., 2020[43]; for DnBA publication in preparation), the formation and elimination of mono-isononyl adipate (MINA) was not further investigated in this study. For DnBA and DEHA, metabolism to the hydrolysis product adipic acid has been shown to be of major importance (DEHA: 10-40 %, DnBA: 14-26 %) (for DnBA publication in preparation; Nehring et al., 2020[43]). Further extensive metabolism resulting in carbon dioxide has been described for DEHA in rats (Takahashi et al., 1981[54]). Therefore, we assume that rapid hydrolysis and elimination via non-specific adipic acid and further break-down to carbon dioxide will be of similar importance also for DINA. Because of the difficulties in adipic acid determination (due to high, fluctuating background levels already known for DEHA and DnBA) we waived the determination of adipic acid in the DINA metabolism samples. 

### DINA exposure assessment in a pilot population

The newly identified, specific DINA metabolites were analyzed in a pilot population from Germany (35 spot urine samples from the general population; all without known DINA exposure). Concentrations of all three DINA metabolites were below the respective LOQ. Nevertheless, the derived F_UE_s enable the calculation of daily intakes (DI) in a worst case scenario, using the LOQ as upper bound metabolite concentrations. We performed the calculation of DIs according to Kohn et al., 2000[35] and Koch et al., 2003[31] as described in Nehring et al., 2020[43] on the example of DEHA. Based on the F_UE_ of the major metabolite OH-MINA worst case DIs for DINA were calculated to be lower than 50 µg/kg bw/d, ranging between 34 and 43 µg/(kg bw/d). Taking into account the DNEL of 850 µg/kg bw/day for DINA (ECHA, 2020[17]), this would indicate that worst case exposures of the pilot population would be at least a factor of 20 below the DNEL (oral route) for the general population. These low (non-detectable) DINA exposures in our (adult) German pilot population are in line with the main sources of DINA exposure expected from toys and childcare articles, as well as from migration from FCM into food (in Europe DINA is not permitted for use in FCM). 

## Conclusion

For the first time, data on human DINA metabolism and urinary excretion were provided. Three monoester metabolites with oxidative modifications (hydroxy, oxo, and carboxylic acid groups) in the isononyl (technical mixture, thus containing several isomers) side chain were identified (OH-MINA, oxo-MINA, and cx-MIOA) as urinary metabolites of DINA. An analytical method for their determination was developed, capturing the sum of isomers of each of the metabolites. The urinary excretion of the oxidized DINA monoester metabolites was quantitatively investigated after oral dose in three volunteers. All three metabolites emerged quickly, reaching peak concentrations at around 2 h post dose. Based on their urinary excretion, urinary excretion fractions (F_UE_s) were derived, which were low (sum of F_UE_s ≤0.034 %), but in a comparable range as those of the two adipates DEHA and DnBA. In analogy to these adipate plasticizers, adipic acid can be expected as a major metabolite of DINA (not investigated). Yet, its lack of specificity in conjunction with high background concentrations renders it useless as exposure biomarker. OH-MINA was the major specific DINA metabolite with inter-individually highly consistent F_UE_s (0.020-0.023). In a pilot population of German adults, DINA monoester metabolite concentrations were below the LOQ. We expect higher DINA exposure in populations from countries which permit the use of DINA in FCM, but also in children due to DINA containing toys and childcare articles; or in occupationally exposed populations. For these populations the above presented DINA metabolites can be used as valuable and specific exposure biomarkers with our methods LOQ sufficiently low enough to be able to sensitively detect potential DNEL exceedances. The newly derived F_UE_s in conjunction with the sensitive analytical HBM method allow to reliably assess DINA exposures and perform risk assessments based on daily intakes. 

## Acknowledgement

The development of the analytical method and its application in investigating human metabolism and population samples are part of a large-scale 10-year project on the advancement of human biomonitoring in Germany. This project is a cooperation agreed in 2010 between the Federal Ministry for the Environment, Nature Conservation, and Nuclear Safety (BMU) and the Verband der Chemischen Industrie e.V. (German Chemical Industry Association- VCI) and is managed by the German Environment Agency (UBA). Experts from governmental scientific authorities, industry and science accompany the project in substance selection and method development (Kolossa-Gehring et al. 2017[36]). The analytical method development was financed by the Chemie Wirtschaftsförderungsgesellschaft mbH.

## Compliance with ethical standards

The study has been reviewed by the Ethics Commission of the medical faculty of the Ruhr University Bochum, Germany (IRB Reg. No.: 15-5422 and 3867-10). The study was performed in accordance with the Code of Ethics of the World Medical Association (1964 Declaration of Helsinki and its later amendments). The study design was presented to the participants in written form and written informed consent was obtained from each participant.

## Conflict of interest

The participation of Rainer Otter as co-author was conducted as part of his employment responsibilities with BASF SE, a manufacturer of DINA, and his advisory role within the BMU-VCI cooperation project on human biomonitoring. The interpretation and views expressed in this manuscript are not necessarily those of the co-author´s employer.

## Supplementary Material

Supplementary material

## Figures and Tables

**Table 1:**
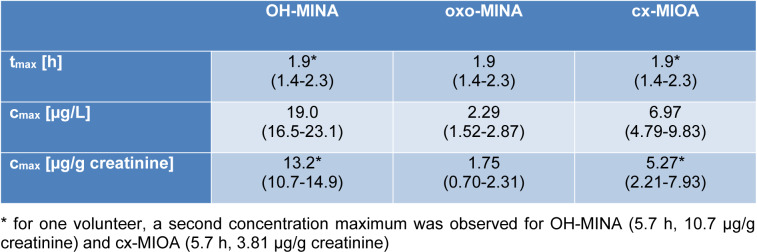
Elimination kinetics for OH-MINA, oxo-MINA, and cx-MIOA in three volunteers after a single oral dose. Peak concentrations (c_max_ - first maximum) after oral dose and their respective time points (t_max_) (mean values; ranges in parentheses). Given the limited number of data points, elimination half-lives were not calculated

**Table 2:**
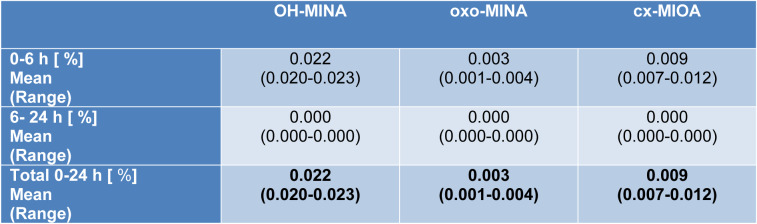
Urinary excretion fractions (F_UE_s) of the DINA metabolites OH-MINA, oxo-MINA, and cx-MIOA after a single oral dose in three healthy volunteers (mean values; ranges in parentheses)
