# Supplementary material for: Quantitative investigation of the urinary excretion of three specific monoester metabolites of the plasticizer diisononyl adipate (DINA)
Source: EXCLI J. 2021 Feb 19;20:412–25. doi: 10.17179/excli2021-3360 (PMC7975632; doi:10.17179/excli2021-3360)
Supplement: Supplementary material [file EXCLI-20-412-s-001.pdf]

EXCLI Journal 2021;20:412-425 – ISSN 1611-2156
Received: January 06, 2021, accepted: February 11, 2021, published: February 19, 2021

Supplementary material to:

Original article:

QUANTITATIVE INVESTIGATION OF THE URINARY EXCRETION OF THREE SPECIFIC MONOESTER METABOLITES OF THE PLASTICIZER DIISONONYL ADIPATE (DINA)

Alexandra Gotthardt[1], Daniel Bury[1], Hans-Willi Kling[2], Rainer Otter[3], Tobias Weiss[1], Thomas Brüning[1], Holger M. Koch[1]*

[1] Institute for Prevention and Occupational Medicine of the German Social Accident Insurance, Institute of the Ruhr-University Bochum (IPA), Bürkle-de-la-Camp-Platz 1, 44789 Bochum, Germany
[2] University of Wuppertal, Department of Chemistry and Biology, Gaußstraße 20, 42119 Wuppertal, Germany
[3] BASF SE, Industrial Petrochemicals Europe; E-CPI/R-H202, Carl-Bosch-Straße 38, 67056 Ludwigshafen, Germany

* **Corresponding author:** Holger M. Koch, Institute for Prevention and Occupational Medicine of the German Social Accident Insurance, Institute of the Ruhr-University Bochum (IPA), Bürkle-de-la-Camp-Platz 1, 44789 Bochum, Germany, Tel.: +49 (0)30 13001 4415, Fax: +49 (0)30 13001 864415, E-mail: koch@ipa-dguv.de

https://orcid.org/0000-0002-8328-2837 (Holger M. Koch)

http://dx.doi.org/10.17179/excli2021-3360/supplementary_material

This is an Open Access article distributed under the terms of the Creative Commons Attribution License (http://creativecommons.org/licenses/by/4.0/).

This supplementary material includes:
Further information on the chemical analyses of the specific DINA (and DEHA) metabolites.

CHEMICAL ANALYSIS

Chromatographic conditions

A 1260 Infinity HPLC system (Agilent Technologies, Waldbronn, Germany) was used, consisting of a G1367E autosampler, a G1311B quaternary pump, a G1312B binary pump coupled with a G4225A vacuum degasser, and a G1316A thermostated column compartment with a 6-port valve. The quaternary pump was connected with the autosampler and was used as loading pump, while the binary pump was connected with the analytical column (via the 6-port valve) for chromatographic separation. The 6-port valve was used to direct the eluent flow from both pumps to operate the two-column assembly. In position A ("loading position") the flow from the quaternary pump was used to transfer the sample from the autosampler, via the 6-port valve, onto the enrichment column and from there (again via 6-port

EXCLI Journal 2021;20:412-425 – ISSN 1611-2156
Received: January 06, 2021, accepted: February 11, 2021, published: February 19, 2021

valve) into the waste. Simultaneously, the flow from the binary pump was directed (via the 6-port valve) onto the analytical column (which was coupled to the MS). For transfer of the analytes from the enrichment column to the analytical column, the valve was switched to position B (“transfer position”). In that position, the flow from the binary pump was directed onto the enrichment column and then onto the analytical column, while the flow from the quaternary pump was diverted directly into the waste. After analyte transfer was complete, the valve was switched back to position A for the chromatographic analysis.

As eluents water (solvent A) and acetonitrile (solvent B), each containing 0.05 % acetic acid, were used for both pumps. The solvent gradients used for analyte enrichment and for chromatographic separation are shown in Supplementary Table 1 and 2. The flow rate on the binary pump was kept at 300 µL/min.

Supplementary Table 1: Solvent gradient for analyte enrichment (quaternary pump)

Time [min]	Flow [µL/min]	Eluent A [%]	Eluent B [%]
**0**	1500	100	0
**3.5**	1500	100	0
**4**	500	100	0
**6**	500	100	0
**12**	500	5	95
**22**	500	5	95
**23**	500	100	0
**24**	1000	100	0
**25**	1500	100	0
**27**	1500	100	0

Supplementary Table 2: Solvent gradient for the chromatographic separation (binary pump)

Time [min]	Eluent A [%]	Eluent B [%]
**0**	75	25
**2.5**	75	25
**3.5**	65	35
**9**	55	45
**14**	5	95
**23**	5	95
**23.5**	75	25
**27**	75	25

The gradient delay volumes according to the manufacturer’s specifications were 600-800 µL for the binary pump flow path and 870-1170 µL for the quaternary pump flow path (including 270 µL gradient delay volume of the autosampler). For analyte enrichment and matrix depletion the switching valve was kept in position A for three minutes. Afterwards, the

EXCLI Journal 2021;20:412-425 – ISSN 1611-2156
Received: January 06, 2021, accepted: February 11, 2021, published: February 19, 2021

6-port valve was switched to position B for analyte transfer and switched back after 2 min (5 min of total runtime) for chromatographic separation and flushing/re-equilibration of the enrichment column. The column compartment was kept at 25±1 °C. The injection process included a needle wash with methanol/water 8:2 (v/v) for 10 s.

Mass spectrometric conditions

An AB Sciex 4500 triple quadrupole mass spectrometer (Darmstadt, Germany) was used for detection. The instrument voltages were -4.5 kV (ion spray voltage), -10 V (entrance potential), and -11 V (collision cell exit potential). Instrument gases (nitrogen) were set to 35 psi (curtain gas), 55 psi (nebulizer gas), 50 psi (heater gas), and 6 arbitrary units (collision gas). The source heater temperature was set to 550 °C. DINA and DEHA metabolites were detected in two separate MRM experiments with detection windows of 120 s (DINA metabolites) and 40 s (DEHA metabolites) and target scan times of 0.3 s. Further scheduled MRM conditions (collision energies (CE) and declustering potentials (DP)) were optimized manually for each standard substance (Supplementary Table 3). In case of DINA metabolites, conditions determined for the single isomeric standard substances were applied to the totality of metabolite isomers present in native urine samples. Analyst 1.6.2 was used for instrument control and MultiQuant 3.0.2 for quantitative data analysis (both Sciex, Darmstadt, Germany).

Supplementary Table 3: Time programmed MRM parameters for each analyte

	t_R [min]	*m/z* precursor ion	*m/z* product ion	DP [V]	CE [eV]
**OH-MINA**					
**quantifier**	11.50	287	83	-80	-25
**qualifier**			127		-20
**$^{13}C_6$-OH-MINA**					
**quantifier**	11.50	293	88	-80	-25
**qualifier**			133		-20
**oxo-MINA**					
**quantifier**	12.66	285	83	-80	-20
**qualifier**			127		-15
**cx-MIOA**					
**quantifier**	11.76	301	83	-80	-30
**qualifier**			145		-23
**$^{13}C_6$-cx-MIOA**					
**quantifier**	11.76	307	88	-80	-30
**qualifier**			173		-25
**5OH-MEHA**					
**quantifier**	10.31	273	83	-80	-23
**qualifier**			127		-16
**$^{13}C_6$-5OH-MEHA**					
**quantifier**	10.31	279	86	-80	-45

EXCLI Journal 2021;20:412-425 – ISSN 1611-2156
Received: January 06, 2021, accepted: February 11, 2021, published: February 19, 2021

Supplementary Table 3 (cont.): Time programmed MRM parameters for each analyte

	t_R [min]	*m/z* precursor ion	*m/z* product ion	DP [V]	CE [eV]
**qualifier**			133		-18
**5oxo-MEHA**					
**quantifier**	11.45	271	83	-80	-20
**qualifier**			127		-15
**$^{13}C_6$-5oxo-MEHA**					
**quantifier**	11.45	277	88	-80	-21
**qualifier**			133		-16
**5cx-MEPA**					
**quantifier**	10.63	287	145	-80	-22
**qualifier**			101		-35
**$^{13}C_6$-5cx-MEPA**					
**quantifier**	10.63	293	151	-75	-21
**qualifier**			88		-35

MRM: multiple reaction monitoring, t_R: retention time, DP: declustering potential, CE: collision energy

Supplementary Figure 1 shows the product ion spectra with postulated fragment structures of the unlabeled single isomer DINA metabolite standards (4-methyloctyladipate with oxidative modifications in position 7 or 8 of the alkyl side chain). Fragments attributable to adipic acid were observed at *m/z* 81, 83, 101, and 127 and have already been described for DEHA (Nehring et al., 2019). A fragment at *m/z* 145 (adipic acid) was only observed for 7cx-MINA. This is in line with observations made for DEHA and DnBA metabolites (Nehring et al., 2019, Ringbeck et al., 2020). Ringbeck et al. (2020) offered an explanation for this divergence in fragmentation behavior: the functionalized side chain of DnBA is likely involved in the fragmentation process resulting in *m/z* 145. They concluded that, based on the negative charge present in the side chain carboxylic acid group, an unstable anhydride intermediate is formed, which can readily break down forming the adipic acid anion at *m/z* 145. For 7cx-MIOA, specific alkyl side chain fragments were observed at *m/z* 173 and *m/z* 127, corresponding to *m/z* 159 and *m/z* 113 for 5cx-MEPA (Nehring et al., 2019). Additionally, the 7cx-MIOA molecule can eliminate water resulting in *m/z* 283, followed by elimination of CO_2 to yield *m/z* 239. All postulated fragments were verified by their accurate masses using Q-Orbitrap-MS (below ±5 ppm; Q Exactive Focus, Thermo Scientific, Bremen, Germany) in ESI negative mode (ion spray voltage 2.5 kV, heater temperature 412 °C, ion transfer capillary temperature 256 °C) at maximum resolution setting (R = 70,000). The instrument gases (nitrogen) were set as follows: sheath gas 47.5 arbitrary units, auxiliary gas 11.25 arbitrary units. The S-Lens RF Level was set to 50 (data not shown). Corresponding $^{13}C_6$-labeled internal standards lead to the same fragments, only with the respectively higher masses (Supplementary Figure 1).

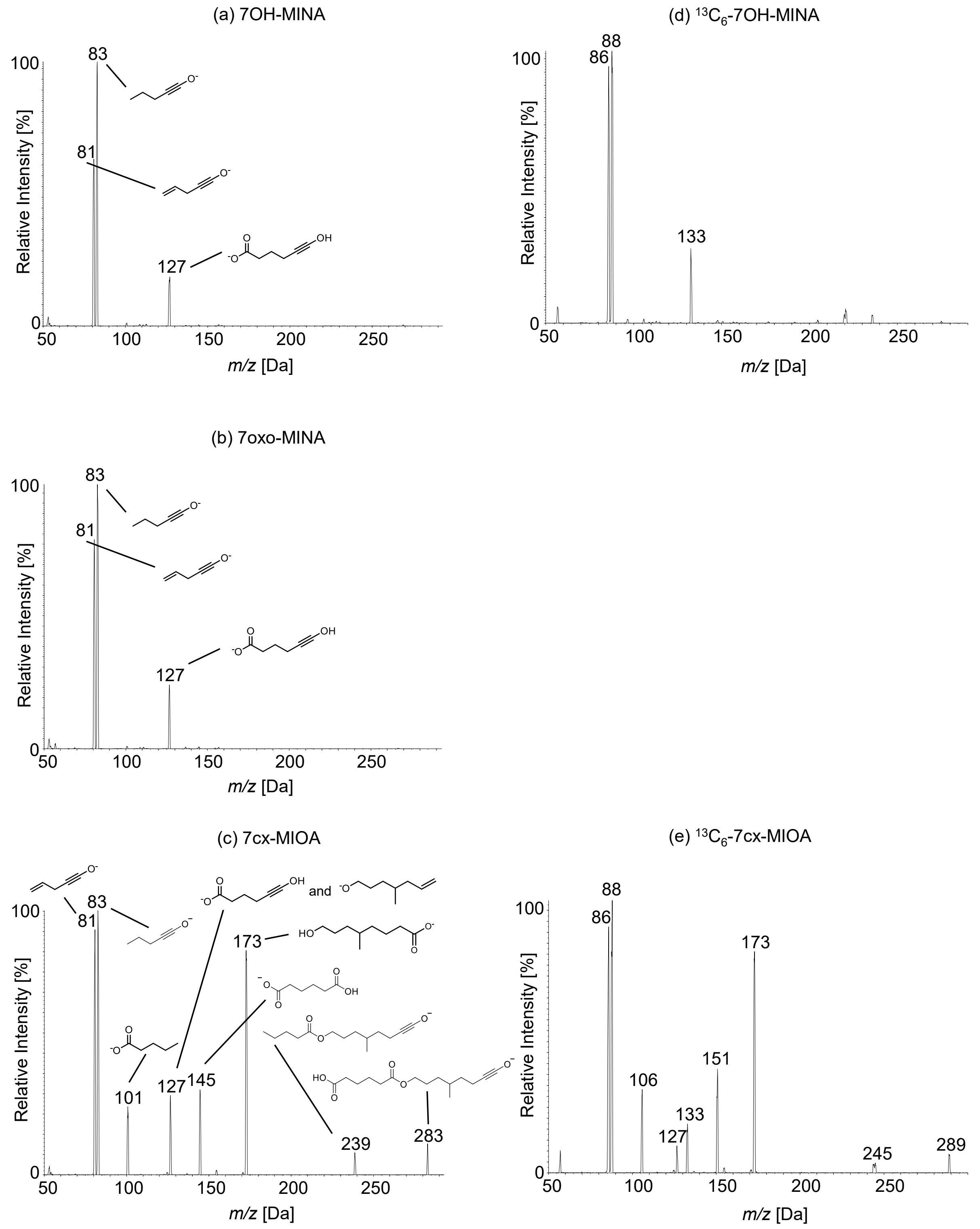

Supplementary Figure 1: Product ion spectra of the unlabeled single isomeric standard substances 7OH-MINA (**a**), 7oxo-MINA (**b**), 7cx-MIOA (**c**), $^{13}C_6$-7OH-MINA (**d**), and $^{13}C_6$-7cx-MINA (**e**) recorded via direct infusion of the individual standard solutions (1 mg/L dissolved in acetonitrile/water 50:50 (v/v), containing 0.05 % acetic acid) at 7 µL/min.

EXCLI Journal 2021;20:412-425 – ISSN 1611-2156
Received: January 06, 2021, accepted: February 11, 2021, published: February 19, 2021

Calibration, validation, and quality control

A stock solution (1 g/L) of each unlabeled DEHA and DINA metabolite standard (7OH-MINA, 7oxo-MEHA, 7cx-MIOA, 5OH-MEHA, 5oxo-MEHA, 5cx-MEPA) and their $^{13}C_6$-labeled analogs (except for 7oxo-MINA, for which an internal standard was not available - instead, $^{13}C_6$-7cx-MIOA was used as a surrogate internal standard) was prepared in acetonitrile. An aqueous internal standard solution containing 150 µg/L of each ^{13}C-labeled standard was prepared from the respective stock solutions. Calibration solutions (containing the non-labeled standards) with concentrations ranging between 0.05 µg/L and 22 µg/L were prepared in water. All solutions were stored in glass flasks, capped with screw caps with silicone/teflon septa until further use. Calibration curves, obtained by weighted (concentration^{-1}) linear regression, were linear throughout the entire calibration range (0.05 µg/L and 22 µg/L) for all metabolites ($r \geq 0.999$). The limit of quantification (LOQ) of each metabolite was estimated based on a signal-to-noise-ratio (S/N) of 10 in native urine samples containing the target analytes. For the multi-isomeric DINA metabolites, the LOQ was attributed to concentrations resulting in an S/N ratio of 10 for the major isomeric peak.

For the determination of method precision and for quality control, quality control material at three different concentration levels (Q_{low}, Q_{med}, and Q_{high}) was prepared by pooling individual spot urine samples containing native DINA and DEHA metabolite concentrations. This material was treated the same way as urine samples and was analyzed in each analytical batch. In the Q_{high} material, DEHA metabolite levels were obtained by spiking (using authentic standards). To determine the precision of the method, Q_{low}, Q_{med}, and Q_{high} were analyzed six times within one series (intra-day precision) as well as on six different days (inter-day precision). Imprecision of the method (both intra- and inter-day) was ≤ 12 % for all analytes (Supplementary Table 4).

Accuracy (in terms of relative recovery) and ruggedness in regard to differences in urinary matrix composition were determined by analyzing eight different urine samples with varying creatinine concentrations (0.3 – 2.3 g/L), both spiked at three different concentration levels (1, 5, and 15 µg/L), as well as without spiking. Relative recoveries were calculated after subtraction of native concentrations measured in the unspiked samples. For oxo-MINA, for which no authentic internal standard was available, in one urine sample relative recoveries around 40 % were observed over all spiking levels, whereas relative recoveries in the remaining samples ranged between 72 and 112 %. For all other metabolites, for which authentic internal standards were available, relative recoveries were between 90 and 121 % (Supplementary Table 5). No relation between creatinine content and relative recoveries was observed for any of the analytes.

EXCLI Journal 2021;20:412-425 – ISSN 1611-2156
Received: January 06, 2021, accepted: February 11, 2021, published: February 19, 2021

Supplementary Table 4: Precision data of the method. All quality control samples (Q_{low}, Q_{med}, Q_{high}) were prepared from urine samples containing native DINA and DEHA metabolite concentrations (except for Q_{high}, in which DEHA metabolites were spiked using analytical standards).

Analyte	Intra-day (n=6)						Inter-day (n=6)					
	Q_{low}		Q_{med}		Q_{high}		Q_{low}		Q_{med}		Q_{high}	
	Mean [µg/L] (range)	RSD [%]	Mean [µg/L] (range)	RSD [%]	Mean [µg/L] (range)	RSD [%]	Mean [µg/L] (range)	RSD [%]	Mean [µg/L] (range)	RSD [%]	Mean [µg/L] (range)	RSD [%]
**OH-MINA**	0.76 (0.73-0.79)	2.9	3.35 (3.30-3.41)	1.2	35.0 (34.5-35.4)	0.8	0.70 (0.64-0.76)	5.8	3.37 (3.23-3.64)	4.2	32.9 (29.6-35.0)	5.5
**oxo-MINA**	<LOQ (<LOQ)	N/A	0.51 (0.49-0.52)	1.9	2.24 (2.20-2.29)	1.6	<LOQ (<LOQ)	N/A	0.51 (0.50-0.55)	3.6	1.94 (1.64-2.24)	12
**cx-MIOA**	<LOQ (<LOQ)	N/A	1.93 (1.79-2.12)	5.9	15.4 (14.9-15.8)	2.7	<LOQ (<LOQ)	N/A	1.84 (1.63-1.94)	6.4	14.4 (13.3-15.6)	7.5
**5OH-MEHA**	<LOQ (<LOQ)	N/A	0.54 (0.53-0.57)	3.8	7.38 (6.97-7.63)	3.4	<LOQ (<LOQ)	N/A	0.53 (0.48-0.58)	6.9	7.28 (6.98-7.58)	7.6
**5oxo-MEHA**	<LOQ (<LOQ)	N/A	0.47 (0.46-0.48)	1.6	7.01 (6.89-7.08)	1.1	<LOQ (<LOQ)	N/A	0.46 (0.44-0.49)	4.5	6.90 (6.70-7.15)	2.3
**5cx-MEPA**	0.48 (0.46-0.49)	2.8	8.07 (7.85-8.25)	2.0	13.9 (13.6-14.1)	1.5	0.46 (0.44-0.49)	4.1	7.81 (7.41-8.35)	4.7	13.4 (12.6-14.2)	4.3

RSD: relative standard deviation

EXCLI Journal 2021;20:412-425 – ISSN 1611-2156
Received: January 06, 2021, accepted: February 11, 2021, published: February 19, 2021

Supplementary Table 5: Accuracy (relative recovery) calculated from analysis of eight different urine samples with different creatinine concentrations (0.34 to 2.3 g/L creatinine) and spiked with approximately 1 µg/L, 5 µg/L, and 15 µg/L

**Analyte**	Native concen-trations [µg/L]	**Low**			**Medium**			**High**		
		Spiked concentra-tions [µg/L]	Measured concentra-tions[a] [µg/L]	Accuracy [%]	Spiked concentra-tions [µg/L]	Measured concentra-tions[a] [µg/L]	Accuracy [%]	Spiked concentra-tions [µg/L]	Measured concentra-tions[a] [µg/L]	Accuracy [%]
**OH-MINA**	<LOQ	0.97	1.06 (0.94-1.12)	109 (97-115)	4.67	4.87 (4.52-5.19)	104 (97-111)	17.5	18.7 (17.0-19.5)	107 (97-111)
**oxo-MINA**	<LOQ	0.97	0.83 (0.44-1.09)	85 (45-112)	4.67	3.66 (1.78-4.73)	78 (38-101)	17.5	14.3 (7.53-18.2)	82 (43-104)
**cx-MIOA**	<LOQ	0.84	0.86 (0.78-0.92)	102 (93-110)	4.03	4.06 (3.79-4.24)	101 (94-105)	15.1	15.9 (14.8-16.7)	105 (98-110)
**5OH-MEHA**	<LOQ	0.89	0.93 (0.85-1.00)	105 (96-112)	4.27	4.17 (3.84-4.40)	98 (90-103)	16.0	16.5 (15.3-17.7)	103 (96-111)
**5oxo-MEHA**	<LOQ	0.86	0.91 (0.86-0.95)	105 (96-110)	4.14	4.29 (4.00-4.51)	104 (97-109)	15.5	16.8 (15.5-17.5)	110 (101-113)
**5cx-MEPA**	<LOQ-0.15	0.77	0.89 (0.82-0.93)	116 (107-121)	3.68	4.10 (3.92-4.34)	111 (106-118)	13.8	16.2 (14.7-16.6)	117 (106-120)

Mean values (ranges in parenthesis) for measured concentrations and accuracies; [a]background levels substracted; LOQ: limit of quantification (OH-MINA and oxo-MINA: 0.3 µg/L, cx-MIOA: 0.6 µg/L, 5OH-MEHA: 0.5 µg/L, 5oxo-MEHA: 0.1 µg/L, and 5cx-MEPA: 0.05 µg/L)

EXCLI Journal 2021;20:412-425 – ISSN 1611-2156
Received: January 06, 2021, accepted: February 11, 2021, published: February 19, 2021

REFERENCES

Nehring A, Bury D, Kling H-W, Weiss T, Brüning T, Koch HM. Determination of human urinary metabolites of the plasticizer di(2-ethylhexyl) adipate (DEHA) by online-SPE-HPLC-MS/MS. J Chromatogr B Analyt Technol Biomed Life Sci. 2019;1124:239–46. doi: 10.1016/j.jchromb.2019.06.019.

Ringbeck B, Bury D, Hayen H, Weiss T, Brüning T, Koch HM. Determination of di-n-butyl adipate (DnBA) metabolites as possible biomarkers of exposure in human urine by online-SPE-LC-MS/MS. J Chromatogr B Analyt Technol Biomed Life Sci. 2020;1141:122029. doi: 10.1016/j.jchromb.2020.122029.
